# Chemometric Analyses for the Characterization of Raw and Stir-Frying Processed Drynariae Rhizoma Based on HPLC Fingerprints

**DOI:** 10.1155/2021/6651657

**Published:** 2021-06-05

**Authors:** Jing Hu, Jiali Wang, Beibei Qin, Lizhi Wang, Xia Li

**Affiliations:** ^1^School of Chinese Materia Medica, Tianjin University of Traditional Chinese Medicine, Tianjin 301617, China; ^2^Tianjin Key Laboratory for Modern Drug Delivery & High-Efficiency, School of Pharmaceutical Science and Technology, Tianjin University, Tianjin 300193, China

## Abstract

The processing of traditional Chinese medicine (TCM) is a necessary practice and usually occurs before most herbs are prescribed. According to Chinese medicine theory, raw (RDR) and stir-frying processed (PDR) Drynariae Rhizoma have different clinical applications. The purpose of this study was to establish HPLC fingerprints coupled with chemometric methods to evaluate the differences between RDR and PDR. Multivariate chemometric methods were applied to analyze the obtained HPLC fingerprints, including hierarchical cluster analysis (HCA), principle components analysis (PCA), and partial least squares discriminant analysis (PLS-DA). The results indicated that RDR and PDR samples showed a clear classification of the two groups, and seven chemical markers having great contributions to the differentiation were screened out. The findings suggested that 5-hydroxymethyl-2-furaldehyde (5-HMF) with a variable importance in the project (VIP > 1) can be used to differentiate between RDR and PDR. Moreover, 5-HMF, naringin, and neoeriocitrin were determined to evaluate their contents in RDR and PDR. The chemometrics combined with the quantitative analysis based on HPLC fingerprint results indicated that stir-frying processing may change the contents and types of components in Drynariae Rhizoma. These changes are probably responsible for the various pharmacological effects of RDR and PDR.

## 1. Introduction

Gu-Sui-Bu (Drynariae Rhizoma, DR) is derived from the dried rhizome of *Drynaria fortunei* (Kunze) J. Sm., which is widely used in China and other Asian countries, for example, Japan, Korea, and Vietnam. According to the “Ben Cao Gang Mu,” the most complete and comprehensive medical literatures concerning TCM, DR has been used in China for thousands of years to tonify kidneys and strengthen bone and subsequently to treat bone fracture, trauma, kidney deficiency, low back pain, muscle weakness, tinnitus, and deafness. Moreover, DR is also the main ingredient of many Chinese patent medicines such as Capsule for Osteoporosis, Pellets for Wound and Wetness, and Decoction for Enrich Blood and Energy [1–4].

According to TCM theory, processing of Chinese Materia Medica is an essential technique, which can improve therapeutic efficacy and reduce toxicity, drastic properties, or side effects. Crude DR products are densely covered with scales, not easy to remove, and their textures are hard and tough, which are not easy to crush and extract effective ingredients. There are two common forms of DR in the medicinal market, including raw DR (RDR) and stir-frying processed DR (PDR). RDR is obtained from dried rhizome of *D. fortunei*, and impurities and ashes are removed. PDR is obtained by stir-frying DR with sand [4, 5]. After stir-frying processing, the drugs are expanded by heat, the textures are crisp, and the villi are scorched and easy to remove, so the drugs are beneficial to decoct effective ingredients.

Chemical analysis has shown that flavonoids, phenylpropanoids, triterpenoids, and phenolic acids are the main compounds isolated from rhizome of *D. fortunei* [6–9]. The total flavonoids from DR were reported to improve bone health, maintain bone mineral density in a low-bone-mass/osteoporosis model system, and activate cyclic AMP response elements [10–12]. Flavonoids such as naringin and neoeriocitrin are the major active ingredients of DR with a wide range of biological activities. Naringin is considered the main effective compound of DR and represents multiple therapeutic targets in bone tissues. Different studies have shown that naringin has a wide range of pharmacological activities, including anti-inflammatory, anticancer activities, as well as effects on bone regeneration, metabolic syndrome, oxidative stress, genetic damage, and central nervous system diseases [13–15]. Neoeriocitrin also significantly increased proliferation of UMR 106 cells and exhibited effects on proliferation and osteogenic cell differentiation in MC3T3-E1 [16, 17]. Furthermore, phenolic acids in DR increased MMP-2 activity and stimulated angiogenesis and cell migration in vivo and in vitro [18]. In addition, drynachromoside A exhibited the biochemical effects on the proliferation of MC3T3-E1 cells [19].

At present, the quality control of RDR and PDR is mainly conducted according to Chinese Pharmacopoeia in which only naringin is determined by the HPLC-UV method [4]. Both RDR and PDR are used clinically, but different medicinal preparations have different requirements. The use of naringin as the unique marker component of quality control for RDR and PDR is not enough, and sometimes it will cause a biased assessment. Then, it is essential to establish methods to discriminate and establish quality control of RDR and PDR.

The efficacy of TCM is the joint action of multicomponents and multitargets, so it is difficult to reflect its integrity by determining a single component or several indexes. Chromatographic fingerprint, based on a systematic research on the chemical composition of analytes, is an effective identification method for comprehensive quality control of TCM. Moreover, the chemical composition, quality, and efficacy of TCM may vary with the growth environment, climatic conditions, harvest time, and processing methods. Meanwhile, fingerprint techniques have been used as a powerful tool in the characterization and authentication of medicinal plants and herbal products [20–22]. Principal component analysis (PCA) and hierarchical cluster analysis (HCA) are useful chemometric models, which make it possible to analyze high-throughput data of samples. The combination of chromatographic fingerprint and chemometrics has been increasingly applied to the characterization and authentication of TCM [23–26].

In this study, HPLC fingerprints of the RDR and PDR were compared, and the fingerprint data sets were applied for classification by several chemometrics methods, such as HCA, PCA, and partial least squares discriminant analysis (PLS-DA). According to the statistical results combined with the chromatographic fingerprints, peaks responsible for discrimination between RDR and PDR were found and how they change in the course of processing was also analyzed. In addition, the changes in the main active components in RDR and PDR were determined.

## 2. Materials and Methods

### 2.1. Materials and Reagents

HPLC grade methanol and acetonitrile were obtained from Sigma-Aldrich (St. Louis, MO, USA). Acetic acid of analytical grade was obtained from Kemiou Co., Ltd. (Tianjin, China). Water was prepared using a Milli-Q system (Millipore, MA, USA). Naringin, neoeriocitrin, and 5-HMF (purity > 98%) were purchased from Shanghai Yuanye Bio-Technology Co., Ltd. (Shanghai, China). Twenty batches of RDR and PDR were collected (Table 1) from different provinces of China and identified as the dried rhizome of *D. fortunei* by Professor Wenyuan Gao of Tianjin University.

### 2.2. Sample Preparation

Samples of RDR and PDR were powdered and passed through a 24-mesh sieve. Then, each sample powder (0.25 g) was weighed accurately and refluxed in 30 mL methanol solution for 3 h. All samples were examined in triplicate. Appropriate amounts of naringin, neoeriocitrin, and 5-HMF reference standards were accurately weighed and prepared to standard solutions.

### 2.3. Chromatographic Analysis

HPLC analysis was performed on a Shimadzu HPLC system (Kyoto, Japan) with a binary pump and a photodiode array detector. An Accurail C_18_ column (5 *μ*m, 4.6 mm × 150 mm) was used for separation at 30°C. The mobile phase was composed of acetonitrile (A) and 0.4% acetic acid in water (B) using a gradient program of 5% A in 0–5 min, 5–30% A in 5–40 min, 30–50% A in 40–55 min, and 50–80% A in 55–65 min. The detection wavelength was set at 283 nm, and the flow rate was 1 mL·min^−1^. A 10 min re-equilibration time was used between HPLC runs. The sample injection volume was 10 *μ*L.

### 2.4. Methodological Evaluation

The method was validated for precision, repeatability, and stability. The relative retention time (RRT) and relative peak area (RPA) were calculated by using naringin as the reference peak. The precision was evaluated by injecting the same sample solution for six times. The relative standard deviation (RSD) for RRT and RPA was less than 1.3% and 1.9%, which indicated good precision of the method. The repeatability was determined by analyzing six independently extracted samples from the same batch of DR. With RSD less than 1.3% for RRT and 2.4% for RPA, the method showed good repeatability. The stability was analyzed by the same sample solution at 0, 2, 4, 8, 12, and 24 h. The RSD for the RRT and RPA was less than 1.4% and 3.0%, indicating that the sample solution was stable within 24 h.

### 2.5. Data Analysis

The data were collected by LabSolutions LC workstation software (version 2010; Kyoto, Japan). The fingerprint similarity was evaluated by Similarity Evaluation System for Chromatographic Fingerprint software (version 2012) [27]. The HCA, PCA, and PLS-DA of samples were performed using SPSS 21.0 (Chicago, USA) and SIMCA software (version 14.1; Beijing, China).

## 3. Results and Discussion

### 3.1. Optimization of the Sample Extraction

In the experiment, the extraction solvent, method, and time were optimized. In ancient clinical practice, DR was mostly taken after water decoction. To extract as many active components as possible from the medicinal materials, the extraction solvents of water, methanol, and ethanol were compared [2, 8]. It was found that methanol extraction showed the largest number of chromatographic peaks, the largest peak area, and stable baseline. Moreover, soaking, ultrasonic, and reflux extraction methods were compared, and the results showed that the efficiency of reflux extraction was higher. The extraction time was further optimized, and the results showed that exhaustive extraction could be achieved when 0.25 g sample powder was extracted with 30 mL methanol by reflux extraction for 3 h.

### 3.2. Similarity Evaluation

**Figure 1:**
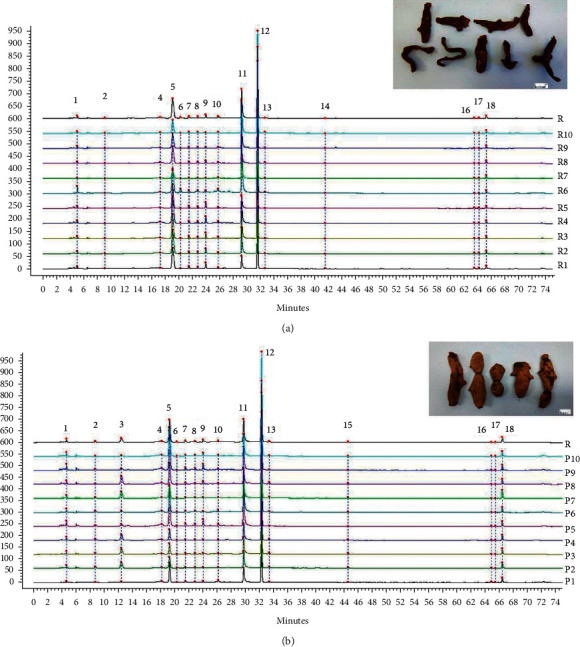
HPLC chromatographic fingerprint of (a) RDR and (b) PDR.

To find the difference before and after processing, the chromatographic fingerprint of RDR and PDR from various sources was evaluated by their similarities. Chromatographic data from 20 batches of RDR and PDR samples were collected, and RDR6 and PDR4 were used as the reference map. The chromatographic fingerprints are shown in Figure 1. The similarity results of RDR and PDR samples were 0.936–0.996 and 0.980–0.995, respectively. The sample information and results are shown in Table 1.

It can be seen that many chromatographic peaks changed to varying degrees. For example, some peak (2, 13, 18) areas increased in PDR, and several peak (6, 8, 9, 16, 17) areas declined obviously. Moreover, peak 14 disappeared after processing. However, some peaks, such as 3 and 15, were present in the PDR.

### 3.3. HCA Modeling

**Figure 2:**
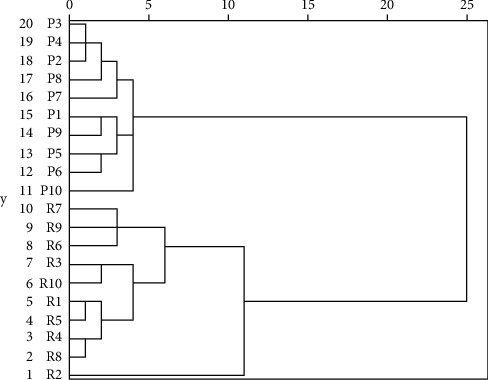
HCA of RDR (R1–R10) and PDR (P1–P10) samples.

HCA is a means of structuring a complex set of observations into unique, mutually exclusive groups (clusters) of subjects similar to each other with respect to certain characteristics [28]. We followed the methods of Cao et al. (2018) [29]. The results of HCA are shown in Figure 2. When the distance level was approximately 12, RDR and PDR samples could be distinguishable. It was evident that RDR and PDR samples were clearly clustered into two groups, which means that the processing procedures caused changes in the composition and/or content of components in DR.

### 3.4. PCA Modeling

**Figure 3:**
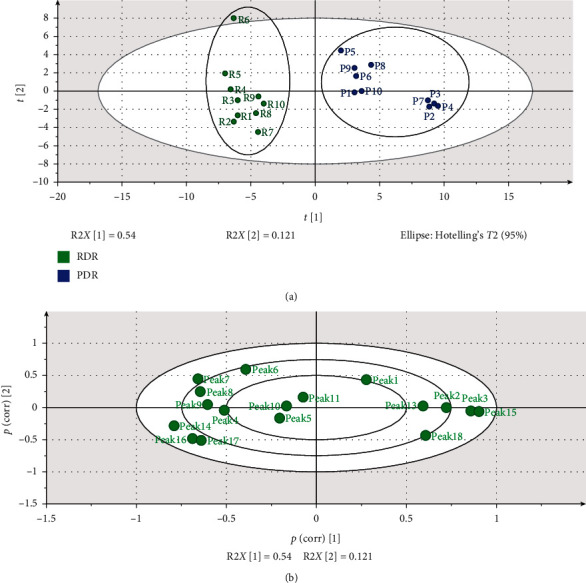
PCA score plot of RDR and PDR samples: (a) scores scatter plot and (b) loading scatter plot.

Principal component analysis (PCA) is an unsupervised bilinear modeling method, which reduces the dimension of data and finds combinations of variables that describe major trends among observations. We followed the methods of Cao et al. (2018) and Zhou et al. (2015) [29, 30]. The first and second principal components (PCs) describe the directions of the two greatest variances in the data and were used to describe RDR and PDR samples. RDR and PDR samples were composed of two separate classes (RDR and PDR), and the results validated the HCA results (Figure 3). Besides, the loading scatter plot shows how the *X*-variables vary in relation to each other, which ones provide similar information and which ones are negatively correlated. It can be easily seen that many variables were responsible for the composition of PCs, among them peaks 3, 14, 15, 16, and 17 featured strongly in identifying RDR and PDR.

### 3.5. PLS-DA Modeling

**Figure 4:**
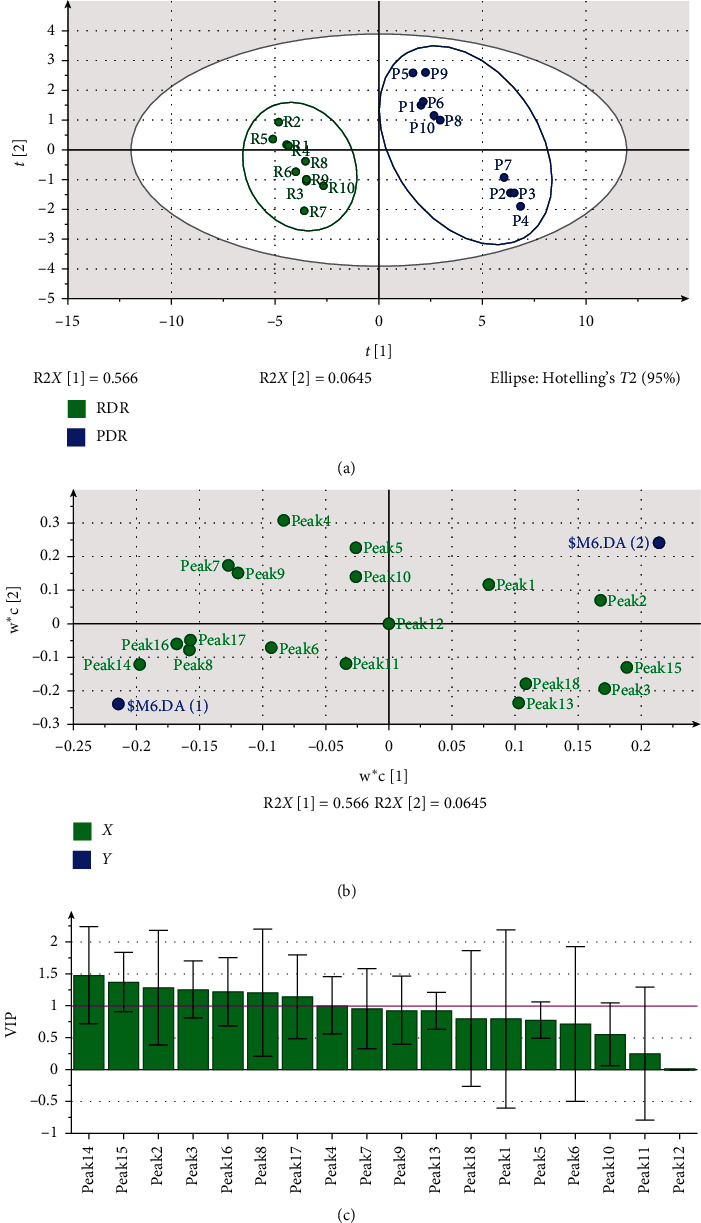
PLS-DA scores plot and VIP (variable importance plot) of DR and PDR samples: (a) scores scatter plot; (b) loading scatter plot; and (c) VIP of peak 1 to peak 18 corresponding to the peaks marked in the chromatogram of HPLC fingerprints. Peaks 2, 3, 8, 14, 15, 16, and 17 were all above 1.0.

As a supervised recognition pattern, PLS-DA can maximize the difference among the groups and aid in the screening of the markers responsible for classification rather than explaining the variations within a data set [31]. In order to find the potential components for the discrimination between the raw and processed samples, PLS-DA was also performed. We followed the methods of Zhou et al. (2015) [28]. First, the raw and processed products could be well distinguished in the scatter plot (Figure 4(a)), indicating that processing plays an important role in the change of DR. Moreover, the samples of RDR clusters were in a small region, while the PDR samples were clustered in a relatively larger range, which illustrated that RDR samples are more stable than PDR samples.

The loading scatter plot was also conducted, which represented the relation between the *X*-variables (18) and the dummy *Y*-variables (2). By default, *X*-variables situated in the vicinity of the *Y*-variables have the highest discriminatory power. As shown in Figure 4(b), variables 2, 3, 8, 14, 15, 16, and 17 stand close to the *Y*-variables and far from the origin, figuring that such variables were strongly responsible for discrimination.

**Figure 5:**
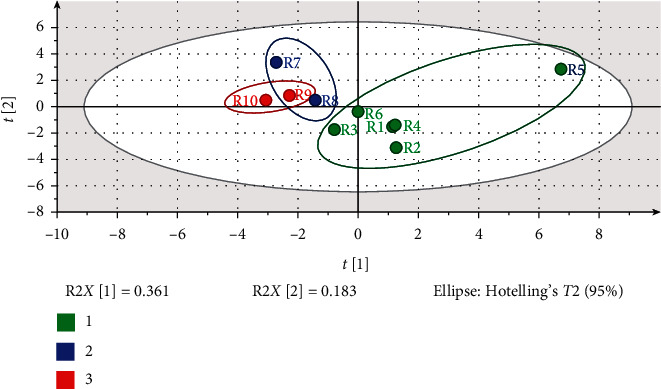
PLS-DA score scatter plot of DR samples collected from different provinces in China: (1) Yunnan; (2) Hunan; and (3) Sichuan. (R2*X* = 0.544, R2*Y* = 0.492, *Q*2 = −0.0301)

Furthermore, in order to weigh the effect of importance of every variable on discrimination, the variable importance for the project (VIP) plot (Figure 5) was carried out, which summarized the importance of the variables both to explain *X* and to correlate to *Y*. According to the VIP plot, some variables (peaks 2, 3, 8, 14, 15, 16, 17) had VIP values larger than 1, which means that these variables were primarily responsible for the discrimination. Among them, 2, 3, 14, and 15 had the largest VIP values, keeping consistent with the analytical result above. With R2*X* = 0.630, R2*Y* = 0.986, and *Q*2 = 0.951, the PLS-DA model was demonstrated to fit the data and predict new data well. Naringin is considered to be the main active constituent of DR, so the quality marker of RDR and PDR is always chosen as naringin [4, 31–33]. Interestingly, the present study indicated that the components significantly influenced by stir-frying processing are peaks 2, 3, 14, and 15. Therefore, the results will provide new ideas for optimizing both stir-frying processing conditions and quality control of DR.

In addition, the score scatter plot in PLS-DA was constructed to evaluate the differences among samples collected from various geographic regions. The results reveal that the samples from different provinces in China could not be clearly distinguished (Figure 5). The samples (R1–R6) from the Yunnan province are mostly in the right quadrant, while the samples (R7–R10) from Sichuan and Hunan provinces are in the left quadrant. The samples (R7 and R8) obtained from the Hunan province appear close to samples (R9 and R10) from the Sichuan province. To a certain extent, the results may be related to the latitudinal location and climates of the source areas.

### 3.6. Determination of Naringin, Neoeriocitrin, and 5-HMF

**Figure 6:**
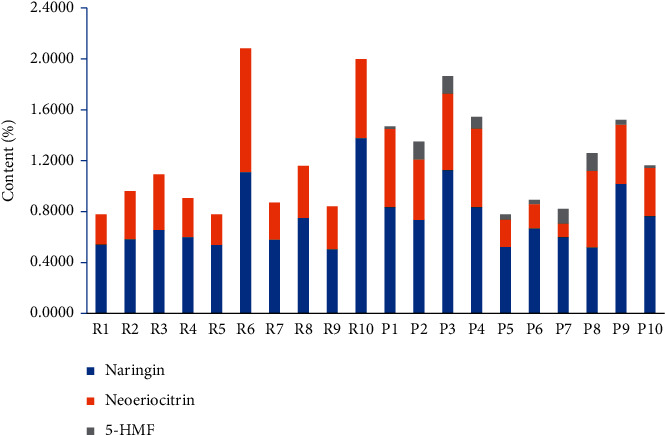
Contents of naringin, neoeriocitrin, and 5-HMF in different batches of RDR and PDR (%). The sample numbers are same as in Table 1.

Peak 3 (5-HMF) with a variable importance in the project (VIP > 1) is the main compound that can be used to differentiate between RDR and PDR. Moreover, peaks 11 (naringin) and 12 (neoeriocitrin) were identified as the main active constituents, the presence of which can be used to differentiate between DR and its related species [34, 35]. The linear regression equations for naringin, neoeriocitrin, and 5-HMF were established by plotting the peak area (*y*) versus concentration (*x*). The regression coefficients (*r*) are >0.999 for the components, indicating a good linearity within a relatively wide range of concentrations. For the precision, the RSD ranged from 0.78% to 0.99%. The samples had good stability at 0, 2, 4, 8, 12, and 24 h, and the RSD (%) ranged from 1.24% to 1.60%. The sample recovery rates of the three components were 99.21∼103.69%, 97.08∼101.24%, and 98.05∼102.02%, respectively. The analyses were analyzed in triplicate, and the results are shown in Table 2 and Figure 6.

As a result, there are both changes in contents and in the composition between RDR and PDR samples. 5-HMF was only detected in PDR products, not in RDR products. 5-HMF was considered a new pharmacological component produced during thermal processing [36]. Studies have demonstrated that 5-HMF inhibited the formation of adipose cells obviously and stimulated the mineralized nodule formation, which indicated that 5-HMF was a powerful inhibitor of adipogenesis and an enhancer of osteoblastogenesis [37]. Besides, 5-HMF improved the morphology of H(2)O(2)-treated human L02 hepatocytes and inhibited the level of caspase-9 and caspase-3 of them [38]. On the other hand, HMF is formed by the degradation of reducing sugars, via the Maillard or caramelization reaction and HMF at high concentrations is cytotoxic [39], which suggests that 5-HMF will help to control the processing parameters of PDR. Moreover, the results indicated that the content of naringin and neoeriocitrin in PDR was higher than RDR to a certain extent. Thus, the expansion of medicinal materials after stir-frying processing may be conducive to the dissolution of the ingredients. However, products from different origins are quite different.

## 4. Conclusions

A novel strategy was established to screen out the potential chemical markers to discriminate RDR and PDR by HPLC fingerprint coupled with multivariate statistical analysis. The results demonstrated that both the unsupervised HCA or PCA and supervised PLS-DA are proved to be satisfactory for separating the samples into two clusters. Seven chemical markers (VIP > 1) were selected for discrimination, and these chemical markers provide a more comprehensive way for the discrimination and quality control of RDR and PDR. Moreover, the content of 5-HMF, naringin, and neoeriocitrin was increased after stir-frying processing. The developed chemometric strategy showed good prospects for the identification and quality control of raw and processed herbal drugs and may also be used for the processing parameters control and investigation of chemical transforming mechanisms underlying the processing.

## Figures and Tables

**Table 1:** Description and similarity evaluation of RDR and PDR batches.

Batches	Similarity	Origin	Source
R1	0.974	Yunnan	RDR
R2	0.936	Yunnan	RDR
R3	0.983	Yunnan	RDR
R4	0.996	Yunnan	RDR
R5	0.973	Yunnan	RDR
R6	0.960	Yunnan	RDR
R7	0.983	Hunan	RDR
R8	0.991	Hunan	RDR
R9	0.990	Sichuan	RDR
R10	0.975	Sichuan	RDR
P1	0.989	Yunnan	PDR
P2	0.995	Yunnan	PDR
P3	0.989	Yunnan	PDR
P4	0.981	Yunnan	PDR
P5	0.991	Yunnan	PDR
P6	0.994	Hubei	PDR
P7	0.984	Guangxi	PDR
P8	0.986	Guangxi	PDR
P9	0.980	Sichuan	PDR
P10	0.987	Sichuan	PDR

**Table 2:** Regression equation, linear range, correlation coefficients (*r*), precision, repeatability, stability, and recovery of naringin, neoeriocitrin, and 5-HMF (*n* = 6).

Compounds	Naringin	Neoeriocitrin	5-HMF
Regression equation	*y* = 1.69 × 10^3^*x* − 1.75 × 10^4^	*y* = 1.74 × 10^7^*x* + 6.27 × 10^4^	*y* = 2.60 × 10^6^*x* − 7.81 × 10^3^
Linear range (*μ*g·mL^−1^)	10∼1000	5.8∼580	2.4–36
*r*	0.9999	0.9997	0.9999
Precision RSD (%)	0.78	0.99	0.88
Repeatability RSD (%)	1.60	1.24	1.12
Stability RSD (%)	0.52	0.32	0.36
Recovery (%)	99.21∼103.69	97.08∼101.24	98.05∼102.02

## Data Availability

The authors have already included a data appointment.
